# Colony-stimulating factor 1 receptor inhibition blocks macrophage infiltration and endometrial cancer cell proliferation

**DOI:** 10.3892/mmr.2020.11197

**Published:** 2020-05-29

**Authors:** Fu Hua, Ye Tian, Yingchun Gao, Changhua Li, Xiaoping Liu

Mol Med Rep 19: 3139-3147, 2019; DOI: 10.3892/mmr.2019.9963

After the publication of the above paper, the authors have realized that the first two affiliations were presented the wrong way around; the Department of Radiotherapy and Oncology, of The Second Affiliated Hospital of Soochow University, Soochow University, should have been listed as the first affiliation on the paper. Therefore, the authors' names and affiliations in this paper should have been presented as follows:

Fu Hua^1,2^, Ye Tian^1,3^, Yingchun Gao^2^, Changhua Li^2^ And Xiaoping Liu^2^

^1^Department of Radiotherapy and Oncology, The Second Affiliated Hospital of Soochow University, Soochow University, Suzhou, Jiangsu 215004; ^2^Department of Gynecology, Huai'an First People's Hospital, Nanjing Medical University, Huai'an, Jiangsu 223300; ^3^Department of Basic Research, Institute of Radiotherapy and Oncology, Soochow University, Suzhou, Jiangsu 215004, P.R. China

The authors regret that this error with the author affiliations was not noticed prior to the publication of their paper, and apologize for any inconvenience caused.

